# Cultural and intergenerational pathways between family alcohol use, trauma exposure, and probable PTSD in Taiwanese adolescents

**DOI:** 10.3389/fpsyt.2026.1752686

**Published:** 2026-06-04

**Authors:** Theodoros Mazarakis, Ching-Yi Kao

**Affiliations:** 1 Department of Counseling & Clinical Psychology, National Dong Hwa University, Hualien, Taiwan; 2 Department of Public Health, Tzu Chi University, Hualien, Taiwan

**Keywords:** adolescent alcohol use, AMIS, Atayal, caregiver drinking, cultural psychiatry, indigenous adolescents, posttraumatic stress disorder (PTSD), trauma exposure

## Abstract

**Background:**

Posttraumatic stress disorder (PTSD) and alcohol use (AU) frequently co-occur, yet little is known about how family drinking patterns shape trauma exposure and PTSD risk in non-Western and Indigenous adolescent populations. Taiwan’s multi-ethnic eastern region—home to Amis and Atayal Indigenous groups—provides a unique context for examining culturally specific trauma pathways.

**Objective:**

To examine how caregiver and adolescent alcohol use relate to exposure to traumatic events (TEs) and probable PTSD likelihood among Han Chinese and two Indigenous groups, Amis and Atayal, in eastern Taiwan.

**Methods:**

A cross-sectional survey was conducted among 751 junior-high students (Han, Amis, Atayal) in rural eastern Taiwan. Adolescents completed the Chinese UCLA PTSD Reaction Index and a trauma-exposure checklist. Caregiver and adolescent AU were assessed via self-report. Logistic regression tested associations among caregiver and adolescent AU, trauma exposure, and probable PTSD likelihood, adjusting for demographic factors.

**Results:**

Caregiver AU was significantly associated with both trauma exposure and probable PTSD across all ethnic groups. While adolescent AU was associated with trauma exposure among Han and Amis youth, no such link was found for Atayal adolescents. Furthermore, exploratory testing rejected the self-medication hypothesis, lending support to the high-risk environmental framework. Trauma types most strongly associated with probable PTSD differed by ethnicity: sexual trauma and painful medical procedures among Han youth; bereavement among Amis adolescents; and witnessing community violence among Atayal adolescents. Indigenous participants showed consistently higher trauma exposure than Han peers.

**Conclusions:**

Family drinking patterns exert strong intergenerational effects on adolescent trauma exposure and probable PTSD risk. The culturally distinct trauma profiles across Han, Amis, and Atayal adolescents highlight the need for trauma-informed, culturally grounded, and family-centered mental-health interventions. Reducing caregiver AU and addressing structural inequities affecting Indigenous communities may offer critical leverage points for preventing PTSD among youth in Taiwan and comparable multi-ethnic settings.

## Introduction

### Global significance of PTSD and alcohol use

Posttraumatic stress disorder (PTSD) is among the most prevalent trauma-related psychiatric conditions worldwide, with lifetime estimates ranging from 5–8% in population-based samples (1). Recent epidemiological syntheses highlight striking cross-national variability in PTSD expression and its developmental correlates, underscoring the importance of cultural and contextual factors (2 3). Beyond individual suffering, PTSD disrupts academic achievement, interpersonal functioning, and broader community well-being. Alcohol-use disorders (AUDs) often co-occur with PTSD and contribute to more severe and persistent psychiatric impairment (4). During adolescence—a developmental period marked by neurobiological plasticity and heightened sensitivity to environmental stressors—PTSD–alcohol comorbidity may form a self-reinforcing cycle: alcohol use (AU) elevates exposure to risky or violent events, while trauma exposure may drive drinking as a means of affect regulation (5, 6).

Three major frameworks have shaped contemporary models of PTSD–alcohol interactions. The self-medication hypothesis proposes that individuals AU to dampen intrusive memories and hyperarousal, inadvertently reinforcing dependence (7, 8). The high-risk pathway posits that early AU increases situational impulsivity and exposure to high-risk settings, thereby elevating trauma likelihood (9). The shared-vulnerability model suggests that PTSD and AUD emerge from overlapping genetic, familial, or environmental liabilities, including caregiver psychopathology, socioeconomic stress, and early adversity (10, 11). While these frameworks are well supported in Western populations, their applicability to East Asian and Indigenous contexts remains insufficiently explored.

### Intergenerational and family pathways

Family environments are central to shaping the developmental trajectories of trauma and substance-use vulnerability. Parental and caregiver AU has been linked to greater childhood trauma exposure, impaired emotion regulation, and elevated risk of adolescent substance misuse (12, 13). Findings from intergenerational trauma research further demonstrate that caregiver substance use may alter children’s neurobiological stress-regulation systems, influence attachment security, and disrupt the predictability of the caregiving environment (7, 14). Within family systems theory, these dynamics operate through multiple pathways—modelled behavior, stress contagion, inconsistent monitoring, and the erosion of protective family structures. Adolescents raised in households where alcohol is normalized or used as a coping strategy may internalize drinking as an acceptable or culturally congruent response to stress, perpetuating risk across generations (15, 16).

### Taiwanese and Indigenous health disparities

Taiwan offers a unique context for examining these developmental and intergenerational processes. The population comprises a Han Chinese majority (≈98%) and 16 officially recognized Indigenous groups of Austronesian origin (17). Indigenous peoples—particularly the Amis and Atayal communities of eastern Taiwan—experience long-standing health inequities, including elevated rates of alcohol-related illness, family violence, and psychological distress (18–20). These disparities reflect structural and historical forces, including colonization, land-dispossession, and socio-economic marginalization, which have contributed to the erosion of traditional kinship systems and community supports (21–25).

Rapid urbanization has further increased Indigenous adolescents’ exposure to commercial alcohol markets and peer drinking environments (26). Despite ongoing public-health efforts, alcohol remains deeply embedded in Indigenous social rituals and family ceremonies. Early informal alcohol initiation—often facilitated by caregivers and occurring despite legal restrictions (27)—creates a dual risk context: higher prevalence of traumatic experiences and greater likelihood of using alcohol as a coping tool (13, 28). Recent studies underscore the importance of cultural determinants of substance use and trauma response, emphasizing how communal norms and collective histories shape the meaning and expression of distress among Indigenous peoples (23, 29).

### Conceptual rationale and study objectives

Much of the literature linking PTSD and AU derives from Western military or clinical samples (30, 31), with comparatively fewer studies examining community-dwelling adolescents in non-Western and Indigenous settings. Cultural values and belief systems influence the interpretation of traumatic events, the visibility of distress, and patterns of help-seeking, underscoring the need for culturally anchored frameworks for understanding PTSD pathways (3, 32). Given the high burden of trauma and AU in Taiwan’s Indigenous communities, investigating how caregiver and adolescent AU interact with culturally patterned trauma exposure is essential for informing trauma-informed and culturally responsive mental-health interventions.

This study addresses these gaps by examining:

1. whether caregiver and adolescent AU is associated with trauma exposure and probable PTSD among Han, Amis, and Atayal adolescents in rural Taiwan; and
2. which trauma types most strongly differentiate PTSD risk across these ethnocultural groups.

Grounded in developmental, cultural, and intergenerational frameworks, we hypothesized that caregiver AU would be associated with PTSD likelihood across all groups, adolescent AU would be more strongly associated trauma exposure among Indigenous youth, and that distinct trauma profiles would characterize each cultural context. By elucidating these pathways, the study aims to advance understanding of culturally embedded mechanisms of risk and to inform targeted, community-grounded interventions for adolescents in Taiwan and comparable multiethnic settings.

## Materials and methods

### Study design and setting

This study used a cross-sectional design within a community-based adolescent mental-health framework. Data were collected between March and May 2012, a period that coincided with the early implementation phase of Taiwan’s Mental Health Act amendment requiring county-level Community Mental Health Centers (33). At this time, national school-based counselling and community mental-health networks were still in early expansion (34). Data were collected from junior-high schools in Hualien County, an eastern coastal region of Taiwan with substantial Amis and Atayal Indigenous populations. The sampling strategy was designed to capture a culturally and geographically diverse adolescent cohort, maintaining representativeness of both Han and Indigenous youth in rural settings.

Hualien’s mountainous terrain and dispersed settlements create significant variability in school size and accessibility. To ensure proportional ethnic representation and reduce cluster bias, six schools were selected using stratified cluster sampling. Both larger schools (>100 students per grade) and smaller schools (≦100 students per grade) were included to mirror the demographic structure of the region.

### Participants

Participants were adolescents aged 13–14 years enrolled in 7th and 8th grades. Of 1,226 students invited, 751 provided both parental and personal written consent and completed all study instruments (response rate = 61.2%). Comparisons between respondents and non-respondents revealed no significant differences in school size, grade distribution, or ethnic composition, although girls were slightly overrepresented in the final sample (54.6% vs. 49.2%).

### Ethical approval and procedure

Ethical approval was granted by the Institutional Review Board of Hualien Tzu Chi Hospital (IRB No. 100-61). Written informed consent was obtained separately from adolescents and caregivers. Survey administration was conducted during regular class periods under teacher supervision. Students were informed that participation was voluntary, that they could withdraw at any time, and that all data would remain anonymous. All questionnaires were coded using non-identifying numeric codes.

Students with symptom scores exceeding the clinical threshold for probable PTSD were discreetly identified, and school counsellors were notified to facilitate appropriate psychological support.

### Measures

#### Demographic variables

Data were collected on age, sex, ethnicity (Han, Amis, Atayal), parental marital status, caregiver type (parents vs. relatives/grandparents), caregiver education (≦9 years vs. > 9 years), and caregiver employment (stable vs. unstable). These variables were selected based on established associations between socioeconomic stability, family structure, and adolescent mental health in Taiwan (21).

#### Alcohol Use of caregivers and adolescents

Adolescents reported both their own and their caregivers’ drinking frequency during the previous year. Caregiver regular drinking was defined as alcohol consumption on two or more occasions per week. Adolescent AU was defined as drinking three or more times in the past year. These operational definitions follow earlier Taiwanese adolescent-health research (13, 28).

#### Traumatic events

Trauma exposure was assessed using the Past Trauma Experience Survey, adapted from the validated Chinese version of the UCLA PTSD Reaction Index for Children and Adolescents (35, 36). The survey includes 13 categories of traumatic events, including natural disasters, traffic accidents, physical assault, sexual trauma, witnessing violence, and painful medical procedures.

Trauma items were categorized as:

• experienced events (e.g., accident, physical assault, sexual trauma, painful treatments), or
• witnessed events (e.g., observing others being injured or assaulted).

#### PTSD symptoms

PTSD symptoms were measured using the 22-item symptom checklist within the UCLA PTSD Reaction Index (UCLA PTSD-RI). Respondents rated symptom frequency from 0 (“none”) to 4 (“most of the time”). Adolescents with a UCLA PTSD-RI score ≧38 were classified as having probable PTSD. Hereafter, this threshold is used to denote individuals at high risk for clinical PTSD symptoms.

The Chinese adaptation has demonstrated strong reliability and validity (α = .92; test–retest r = .80) in Taiwanese adolescent populations (37).

### Data analysis

All analyses were conducted using SPSS version 22.0 (IBM Corp.). Welch’s ANOVA was used to examine group differences in continuous variables due to unequal sample sizes, while Chi-square (χ²) tests assessed differences in categorical variables.

Logistic regression models estimated: 1. the associations between caregiver/adolescent alcohol use and the likelihood of reporting any traumatic event (TE), and 2. the associations between alcohol use and probable PTSD among adolescents with at least one TE.

All models were adjusted for sex, age, caregiver education, and caregiver employment. Statistical significance was set at p <.05 (two-tailed).

### Justification for using 2012 data

Although collected in 2012, the dataset captures a critical transitional moment in Taiwan’s mental-health system. The 2007 amendment of the Mental Health Act initiated the establishment of county-level Community Mental Health Centers, marking a national shift toward community-based and preventive mental-health care (33). Between 2010 and 2013, the Ministry of Health and Welfare began expanding school-based counselling services and community mental-health networks, but nationwide implementation had not yet occurred (34). The 2012 data therefore reflect adolescent trauma exposure, alcohol-use behavior, and PTSD risk prior to large-scale national reform, providing a meaningful baseline for evaluating long-term, structural determinants of adolescent mental health in rural and Indigenous communities.

## Results

### Trauma prevalence and demographic characteristics

Among the 751 adolescents, 59.7% reported at least one traumatic event. Trauma exposure differed significantly by ethnicity: Amis adolescents showed the highest prevalence (69.3%), followed by Atayal (60.0%) and Han youth (51.0%) (**Table 1**). Probable PTSD (UCLA-PTSD-RI ≧38) was identified in 5.7% of the total sample, with the highest proportion found among Amis adolescents (8.3%), compared with Han (6.1%) and Atayal (3.1%).

**Table 1:** Demographic characteristics, trauma experiences, and alcohol–use patterns of adolescents by ethnicity.

All (N = 751)								With trauma (n=448)							
Han Chinese		Amis		Atayal		Total		Han Chinese		Amis		Atayal		Total	
(n=263)		(n=228)		(n=260)		(n=751)		(n=134)		(n=158)		(n=156)		(n=448)	
n	%	n	%	n	%	n	%	n	%	n	%	n	%	n	%
Reporting trauma															
134	51.0**^a b^**	158	69.3 **^c^**	156	60.0	448	59.7								
Probable PTSD likelihood															
16	6.1	19	8.3 **^c^**	8	3.1	43	5.7								
Parents’ marital status–married^¥^															
172	65.4	139	61.0	159	61.2	471	62.6	83	61.9	95	60.1	100	64.1	278	62.1
Primary caregiver–parent															
216	82.1	176	77.2	207	79.6	599	79.8	104	77.6	120	75.9	120	76.9	344	76.8
Caregiver’s education– >9 years															
131	49.8**^a b^**	79	34.6	94	36.2	304	40.5	70	52.2**^a b^**	59	37.3	55	35.3	184	41.1
Caregiver employment–stable															
169	64.3**^a b^**	104	45.6	122	46.9	395	52.6	80	59.7	76	48.1	82	52.6	238	53.1
Caregiver’s drinking–regular^§^															
97	36.9	104	45.6	94	36.2	295	39.3	56	41.8	80	50.6	68	43.6	204	45.5
Self-drinking–drinker^#^															
35	13.3**^a^**	55	24.1**^c^**	20	7.7	110	14.6	26	19.4 **^a^**	49	31.0 **^c^**	10	6.4	85	19.0

**^a^**significant difference between Han and Amis, *p* <.05, **^b^**between Han and Atayal, **^c^**between Amis and Atayal; **^￥^**including cohabited; **^§^**drinking more than twice a week in the past year**; ^#^**drinking more than 3 times in the past year. Bold superscript letters denote a statistically significant difference between groups (a = between Han and Amis, b = between Han and Atayal, c = between Amis and Atayal).

Several socioeconomic indicators varied across groups. Han adolescents had higher proportions of caregivers with >9 years of education (49.8%) compared with both Indigenous groups (Amis 34.6%; Atayal 36.2%). Stable caregiver employment was also more common among Han families (64.3%) than among Amis (45.6%) or Atayal (46.9%) households. Adolescent alcohol use differed markedly, being most prevalent among Amis youth (24.1%), followed by Han (13.3%) and Atayal (7.7%). These patterns were amplified in the trauma-exposed subsample: nearly one-third of Amis adolescents with trauma (31.0%) reported drinking in the past year, compared with 19.4% of Han and 6.4% of Atayal adolescents.

### Associations between AU and trauma exposure

Caregiver regular drinking was associated with increased odds of trauma exposure among Amis adolescents (OR = 2.22, 95% CI: 1.20–4.10, *p* = 0.010) and Atayal adolescents (OR = 2.25, 95% CI: 1.29–3.92, *p* = 0.004), although not among Han youth (OR = 1.55, *p* = 0.095) after adjusting for demographic covariates (**Table 2**).

**Table 2:** Logistic regression analysis of caregiver and adolescent alcohol use predicting trauma exposure among Taiwanese adolescents.

	Han Chinese (n=263)		Amis (n=228)		Atayal (n=260)	
Predictor	OR*(95% CI)	*P*	OR (95% CI)	*P*	OR (95% CI)	*P*
Caregiver’s drinking(reference group: irregular drinker^†^)						
	1.55 ^i^ (0.92–2.61)	0.095	2.22 ^ii^ (1.20–4.10)	0.010	2.25 ^iv^ (1.29–3.92)	0.004
Self-drinking(reference group: non drinker^§^)						
	3.45 ^i^ (1.53–7.80)	0.003	4.74 ^ii^ (1.91–11.77)	0.001	0.57 ^iii^ (0.22–1.46)	0.243

*, OR, odds ratio, CI, confidence interval; ^†^, drinking less than twice a week in the past year; ^§^, drinking less than 3 times in the past year; ^i^, the logistic model controlling for caregiver’s type (CT), education (EDU) and employment status (ES); ^ii^, controlling for parent’s marital status (MS), CT and ES; ^iii^, controlling for MS, CT, EDU and ES; ^iv^, controlling for CT, EDU, and ES.

Adolescent drinking showed similarly patterned associations. Among Han adolescents, drinking increased the likelihood of trauma exposure more than threefold (OR = 3.45, 95% CI: 1.53–7.80, *p* = 0.003). Among Amis adolescents, the association was even stronger (OR = 4.74, 95% CI: 1.91–11.77, *p* = 0.001). No significant association was observed among Atayal adolescents (OR = 0.57, *p* = 0.243), suggesting culturally distinctive pathways linking AU and trauma exposure.

### Associations between AU and probable PTSD among those with trauma

Among trauma-exposed adolescents (n = 448), caregiver drinking was significantly associated with increased probable PTSD likelihood in all ethnic groups, with particularly elevated risk among Atayal adolescents (OR = 10.70, 95% CI: 1.14–99.98, *p* = 0.038) and Han adolescents (OR = 6.12, 95% CI: 1.75–21.38, *p* = 0.004). While OR in Atayal is statistically significant, the expansive confidence interval reflects limited numerical stability, stemming from the low frequency among Atayal participants. Amis adolescents also showed increased risk (OR = 3.78, 95% CI: 1.15–12.33, *p* = 0.028) (**Table 3**). In contrast, adolescent drinking was not significantly associated with probable PTSD in any ethnic group once trauma exposure had occurred. This suggests that caregiver AU—rather than adolescent drinking—emerged as a more salient familial correlate of sustained post-traumatic symptoms.

**Table 3:** Logistic regression analysis of caregiver and adolescent alcohol use predicting probable PTSD among trauma–exposed Taiwanese adolescents.

	Han Chinese (n=263)		Amis (n=228)		Atayal (n=260)	
Predictor	OR*(95% CI)	*P*	OR (95% CI)	*P*	OR (95% CI)	*P*
Caregiver’s drinking(reference group: irregular drinker^†^)						
	6.12 ^i^ (1.75–21.38)	0.004	3.78 ^i^ (1.15–12.33)	0.028	10.70 ^i^ (1.14–99.98)	0.038
Self-drinking(reference group: non drinker^§^)						
	1.36 ^i^ (0.38–4.91)	0.631	0.54 ^ii^ (0.16–1.75)	0.306	1.22 ^i^ (0.11–13.70)	0.868

*, OR, odds ratio, CI, confidence interval; ^†^, drinking less than twice a week in the past year; ^§^, drinking less than 3 times in the past year; ^i^, the logistic model controlling for parent’s marital status (MS), caregiver’s type, education, and employment status (ES); ^ii^, controlling for MS and ES.

### Trauma types and the likelihood of probable PTSD

Across all trauma-exposed adolescents, the most frequently reported trauma types were accidents (50.4%), witnessing family violence (46.7%), painful medical treatment (35.0%), and physical assault (30.8%) (**Table 4**). Trauma profiles varied by ethnicity: accidents were particularly common among Atayal adolescents (56.4%), whereas witnessing community violence was most frequent among Amis (36.7%) and Atayal youth (41.0%).

**Table 4:** Logistic regression analysis of trauma types predicting probable PTSD among trauma–exposed Taiwanese adolescents.

Han Chinese		Amis		Atayal		Total		Han Chinese		Amis		Atayal	
(n=134)		(n=158)		(n=156)		(n=448)							
n	%	n	%	n	%	n	%	OR*/^¶^ (95% CI)	*P*	OR (95% CI)	*P*	OR (95% CI)	*P*
Natural disasters													
39	29.1	35	22.2	38	24.4	112	25.0	1.19 (0.35–4.09)	0.773	0.97 (0.24–3.88)	0.974	1.21 (0.18–7.87)	0.837
Accidents													
57	42.5	81	51.3	88	56.4	226	50.4	0.61 (0.17–2.17)	0.448	0.81 (0.29–2.27)	0.692	1.30 (0.19–8.93)	0.783
Physical assault													
44	32.8	53	33.5	41	26.3	138	30.8	2.01 (0.60–6.72)	0.257	1.53 (0.55–4.23)	0.413	3.15 (0.48–20.74)	0.231
Witnessing family members being physically assaulted													
61	45.5	73	46.2	75	48.1	209	46.7	2.90 (0.84–9.93)	0.089	0.40 (0.13–1.17)	0.096	0.69 (0.11–4.36)	0.700
Sexual trauma													
42	31.3	51	32.3	44	28.2	137	30.6	5.41 (1.51–19.35)	0.009	0.90 (0.29–2.76)	0.859	20.76 (1.77–242.42)	0.016
Witnessing others in community being physically assaulted													
41	30.6	58	36.7	64	41.0	163	36.4	1.72 (0.54–5.49)	0.358	1.20 (0.43–3.34)	0.717	16.66 (1.41–195.65)	0.025
Seeing dead bodies													
21	15.7	28	17.7	19	12.2	68	15.2	2.54 (0.61–10.60)	0.200	.493 0.57 (0.12–2.78)	0.493	1.79 (0.23–13.59)	0.572
Death or severe injury of significant others													
22	16.4	35	22.2	30	19.2	87	19.4	1.48 (0.38–5.75)	0.566	4.16 (1.41–12.21)	0.009	3.04 (0.53–17.23)	0.209
Painful medical treatment													
43	32.1	52	32.9	62	39.7	157	35.0	4.76 (1.42–15.94)	0.011	1.61 (0.57–4.57)	0.364	4.38 (0.54–35.33)	0.165

*OR, odds ratio; CI, confidence interval; ^¶^, the logistic model controlling for parent’s marital status, caregiver’s type, education, employment status and drinking.

Several trauma types were significantly associated with probable PTSD, with patterns differing sharply across ethnic groups:

• Han adolescents showed elevated risk of probable PTSD following sexual trauma (OR = 5.41, *p* = 0.009) and painful medical experiences (OR = 4.76, *p* = 0.011).
• Amis adolescents showed increased likelihood of probable PTSD following the death or severe injury of significant others (OR = 4.16, *p* = 0.009).
• Atayal adolescents showed strikingly high risk of probable PTSD following sexual trauma (OR = 20.76, p = .016) and witnessing community violence (OR = 16.66, *p* = 0.025). Despite the significant p-value, the resulting wide confidence interval indicates a degree of estimate instability due to the sparse cell sizes for specific trauma types.

These findings indicate that the trauma–probable PTSD relationship is culturally patterned, with different trauma types conferring heightened risk in different ethnocultural contexts.

### Exploratory bidirectional analyses: testing the self-medication hypothesis

To address the potential bidirectional relationship between adolescent alcohol use and traumatic stress, two exploratory logistic regression models were conducted to test the “self-medication” pathway, using the same demographic and caregiver covariates employed in the primary analyses. In the first exploratory model, trauma exposure was used as a predictor for adolescent AU across the three ethnic groups (**Supplementary Table 1**). Results indicated that trauma exposure significantly increased the odds of drinking among Han Chinese (OR = 3.21, 95% CI: 1.44–7.15, *p* = 0.003) and Amis adolescents (OR = 4.71, 95% CI: 1.90–11.66, *p* = 0.001). However, similar to the findings in the primary model (**Supplementary Table 2**), this association was not statistically significant for Atayal adolescents (OR = 0.56, 95% CI: 0.21–1.44, *p* = 0.230), suggesting that the link between trauma and alcohol initiation may be buffered by distinct cultural or community factors in this group.

A second model was conducted among trauma-exposed adolescents (n = 448) to determine if the severity of psychological distress (indicated by probable PTSD status) functioned as a driver for “self-medication” through AU (**Supplementary Table 2**). In contrast to the robust findings observed in the “high-risk” pathway (where alcohol predicted trauma exposure), probable PTSD status was not significantly associated with adolescent drinking in any of the three ethnic groups: Han Chinese (*p* = 0.643), Amis (*p* = 0.360), or Atayal (*p* = 0.654).These findings suggest that while trauma and alcohol are concurrently linked among Han and Amis youth, the “self-medication” pathway—whereby adolescents AU specifically to alleviate posttraumatic symptoms—may not yet be established in this early adolescent cohort (ages 13–14). Instead, the data more strongly support a “high-risk” framework, where AU serves as a marker for environmental risk and subsequent trauma exposure rather than a reactive coping mechanism for established PTSD symptoms.

## Discussion and conclusion

### Overview

This study examined how caregiver and adolescent AU relate to trauma exposure and posttraumatic stress among Han, Amis, and Atayal adolescents in rural Taiwan. Three consistent patterns emerged. First, caregiver AU was a robust correlate of both trauma exposure and probable PTSD across all ethnic groups, supporting intergenerational vulnerability mechanisms reported in prior literature (8, 13). Second, adolescent drinking was linked to increased trauma exposure primarily among Han and Amis youth, consistent with high-risk behavioral pathways (15). Third, distinct trauma types were associated with probable PTSD across ethnic groups, underscoring the cultural shaping of trauma meaning and impact (3, 32). These findings highlight the need for culturally grounded, family-centered interventions in multi-ethnic and Indigenous settings.

### Family and intergenerational pathways

Caregiver AU demonstrated a strong and consistent association with both trauma exposure and probable PTSD. This aligns with decades of evidence linking parental substance use, family instability, and youth psychopathology (9, 38). Alcohol-affected households often experience increased conflict, reduced supervision, and disrupted emotional availability, all of which are related to a higher likelihood of children’s exposure to potentially traumatic events (11). Emerging evidence also indicates that trauma and substance use may reinforce one another across generations through digital, social, and behavioral pathways, with caregiver functioning exerting long-term influence on adolescent coping and risk behaviors (16).

Importantly, caregiver drinking remained predictive even after controlling for socioeconomic variables, suggesting a deeper disruption of what has been described as a ‘natural wellbeing’ established at birth. As proposed by Human Birth Theory (39), chronic trauma and early adverse events can impair a caregiver’s primary mental vitality, potentially driving the use of alcohol as a maladaptive self-medication strategy to manage the loss of this innate equilibrium. This framework helps explain how alcohol-affected households become environments of heightened risk, as the erosion of caregiver functioning is inextricably linked to the adolescent’s own developmental stability within the shared family environment. Neuro-developmental research further demonstrates that chronic family stress and parental substance use can dysregulate stress-response systems, including the hypothalamic–pituitary–adrenal axis, increasing vulnerability to PTSD following trauma (7, 14). Taken together, these findings support a multilevel intergenerational transmission model involving behavioral, emotional, and biological pathways.

### Adolescent AU and trauma exposure

Adolescent drinking was significantly associated with trauma exposure among Han and Amis youth but not Atayal adolescents. This pattern aligns with the high-risk hypothesis, which describes a pathway where early AU relates to greater situational impulsivity and exposure to high-risk situations (6, 15). Cultural differences between Indigenous groups may account for the divergent patterns. Atayal communities traditionally maintain kinship structures and moral systems—such as the Atayal *gaga*—discouraging unsupervised youth drinking (40), potentially buffering adolescents from risk. The buffering effect can be further understood through the Developmental Assets model (41). In this context, the Atayal *gaga* may function as critical external assets that provide boundaries and social supervision. By fostering these cultural assets, community-grounded interventions may effectively shield adolescents from initiating high-risk behaviors, even when familial stressors such as caregiver drinking are present.

Genetic differences in alcohol metabolism may contribute additional nuance. The ALDH2 rs671 variant, common among Han Chinese, heightens physiological sensitivity to alcohol (42). This may interact with cultural norms to influence drinking patterns, social settings in which drinking occurs, and associated risks. Notably, adolescent drinking did not predict probable PTSD among trauma-exposed youth, suggesting that alcohol plays a more important role in exposure to traumatic events than in post-trauma coping during early adolescence. This contrasts with self-medication models observed more commonly in adults (31).

### Cultural mediation of trauma and probable PTSD

The trauma types most associated with probable PTSD differed sharply across ethnic groups, reinforcing that trauma is interpreted, experienced, and processed within cultural frameworks (3).

For Han adolescents, sexual trauma and painful medical procedures were the strongest predictors of probable PTSD. These findings are consistent with research suggesting that medically invasive procedures may be particularly distressing in cultural contexts emphasizing bodily integrity, emotional restraint, and high academic expectations (43).

For Amis adolescents, bereavement emerged as the principal predictor. Amis cultural life centers around extended kinship, collective labor, and ritualized mourning (44); sudden loss may therefore destabilize both emotional attachment and communal identity.

For Atayal adolescents, witnessing community violence carried especially high probable PTSD risk. Within the Atayal moral system of gaga, community harmony and reciprocity are foundational; violence represents a profound violation of moral and social order, amplifying its psychological impact.

These culturally differentiated pathways affirm that PTSD cannot be understood without recognizing the sociocultural meanings attached to trauma.

### Indigenous inequality and historical context

Indigenous adolescents consistently showed higher trauma exposure and distinct trauma–probable PTSD pathways. These disparities reflect longstanding structural inequalities in Taiwan, including colonization, economic marginalization, and unequal access to services (19, 23). AU in Indigenous communities is often misinterpreted as an individual behavior rather than a symptom of intergenerational trauma and cultural disruption. The elevated trauma burden observed here underscores the need for trauma-informed, culturally safe mental-health services that acknowledge historical context and Indigenous knowledge systems.

### Public-health and clinical implications

The findings support several intervention priorities:

• Family-based prevention. Routine screening for caregiver drinking in school, community, and primary-care settings may help identify families at elevated risk for both trauma exposure and PTSD.
• Culturally tailored trauma-informed care. Effective services in Indigenous contexts should integrate cultural practices, communal healing, and Indigenous health workers to enhance trust and acceptability (29).
• Targeted responses to trauma type. Bereavement support for Amis youth, community-violence prevention for Atayal communities, and improved communication around medical procedures for Han adolescents may increase clinical relevance and responsiveness.

In addition, to move beyond a purely deficit-focused approach to Indigenous mental health, it is essential to employ a ‘developmental imagination’ (45). This perspective encourages clinicians and policymakers to view Indigenous adolescent trajectories not as deterministically fixed by past trauma, but as dynamic processes with significant potential for restoration. By recognizing the interaction between individual growth and cultural context, we can develop interventions that leverage Indigenous knowledge systems to rebuild healthy developmental pathways.

### Limitations

Several limitations must be acknowledged. First, the cross-sectional design precludes causal inference and limits the ability to establish causality between AU, trauma exposure, and posttraumatic symptoms. While regression models utilize ‘predictors’ and ‘outcomes,’ these should be interpreted as measures of statistical association within a single point in time rather than evidence of a chronological causal chain. Consequently, the observed associations should not be interpreted as definitive causal pathways. Second, while the UCLA PTSD-RI is a validated screening tool, it provides a measure of probable PTSD based on symptom thresholds rather than a confirmed clinical diagnosis. Third, alcohol use was assessed via frequency-based self-reports consistent with prior Taiwanese research, yet the absence of measures for consumption quantity or binge drinking limits the construct validity and precision of this variable. Moreover, frequency alone may not fully distinguish between experimental, low-risk drinking and heavy episodic use, which is often more strongly linked to traumatic injury and PTSD. Consequently, our findings may represent a conservative estimate of the association between alcohol and trauma, as we were unable to isolate the specific effects of high-intensity drinking patterns.

Fourth, the treatment of sexual trauma as a single aggregated category resulted in statistical imprecision and potential model instability in some subgroups, as evidenced by very large odds ratios and wide confidence intervals. Fifth, our analytical model was primarily unidirectional, focusing on how alcohol use predicts trauma and PTSD. This leaves unexamined the bidirectional relationship—specifically the “self-medication” pathway where trauma exposure or posttraumatic symptoms may drive subsequent alcohol use. Sixth, unmeasured factors such as parental mental illness, genetic variants beyond ALDH2, or culturally specific traumas such as discrimination or community conflict may partially account for the observed associations and warrant investigation in future longitudinal studies.

Finally, although the dataset was collected in 2012, its relevance is sustained by recent national statistics from the Ministry of Health and Welfare (46) and the Child Welfare League Foundation (47) which indicate that adolescent suicide rates have reached 18.4%, with past trauma remaining a significant predictor of distress. Furthermore, the chronic 8-year life expectancy gap for Indigenous populations highlights that the structural and familial risk factors examined in this study—specifically alcohol-related pathways—remain a critical, unaddressed frontier in Taiwan. This is further supported by National Health Insurance (NHI) reports from 2024–2025, which highlight that alcohol-related illnesses and mortality continue to be disproportionately high in regions with large Indigenous populations.

## Conclusion

Caregiver alcohol use is a consistent and powerful predictor of trauma exposure and probable PTSD risk among adolescents across Taiwan’s major ethnocultural groups. Yet the trauma types most closely associated with probable PTSD differ by culture, emphasizing the necessity of incorporating cultural interpretation, family dynamics, and structural inequities into trauma-informed adolescent mental-health interventions. Effective policy in Taiwan and comparable societies must prioritize caregiver well-being, culturally grounded prevention, and meaningful partnership with Indigenous communities.

## Data Availability

The datasets presented in this article are not readily available because they contain sensitive information regarding adolescent trauma and alcohol use. Requests to access the datasets should be directed to the corresponding author and are subject to institutional ethical review and data sharing agreements.

## Supplementary material

Supplementary Table 1 Logistic regression analysis of trauma exposure predicting adolescent alcohol use among Taiwanese adolescents. *= OR, odds ratio, CI, confidence interval; ^i^=the logistic model controlling for caregiver’s type (CT), education (EDU) and employment status (ES); ^ii^= controlling for parent’s marital status (MS), CT and ES; ^iii^= controlling for MS, CT, EDU and ES.

Supplementary Table 2 Logistic regression analysis of probable PTSD predicting adolescent alcohol use among trauma–exposed Taiwanese adolescents. *= OR, odds ratio, CI, confidence interval; ^i^=the logistic model controlling for parent’s marital status (MS), caregiver’s type, education, and employment status (ES); ^ii^= controlling for MS and ES.
