# Supplementary material for: Cultural and intergenerational pathways between family alcohol use, trauma exposure, and probable PTSD in Taiwanese adolescents
Source: Front Psychiatry. 2026 Jun 4;17:1752686. doi: 10.3389/fpsyt.2026.1752686 (PMC13275686; doi:10.3389/fpsyt.2026.1752686)
Supplement: Supplementary Table 1 — Logistic regression analysis of trauma exposure predicting adolescent alcohol use among Taiwanese adolescents. *= OR, odds ratio, CI, confidence interval; i=the logistic model controlling for caregiver’s type (CT), education (EDU) and employment status (ES); ii= controlling for parent’s marital status (MS), CT and ES; iii= controlling for MS, CT, EDU and ES. [file SupplementaryFile1.docx]

# Tables

## Table S1. Logistic regression analysis of trauma exposure predicting adolescent alcohol use among Taiwanese adolescents

|  | **Han Chinese (n=263)** | | **Amis (n=228)** | | **Atayal (n=260)** | |
| --- | --- | --- | --- | --- | --- | --- |
| **Predictor** | **OR*(95% CI)** | ***P*** | **OR (95% CI)** | ***P*** | **OR (95% CI)** | ***P*** |
| **Trauma exposure**  **(reference group: no exposure)** | | | | |  |  |
|  | 3.21 **^i^** (1.44–7.15) | 0.003 | 4.71 **^ii^** (1.90–11.66) | 0.001 | 0.56 **^iii^** (0.21–1.44) | 0.230 |

*Note:* *= OR, odds ratio, CI, confidence interval; **^†^=** drinking less than twice a week in the past year**; ^§^=** drinking less than 3 times in the past year**;** ^i^=the logistic model controlling for caregiver’s type (CT), education (EDU) and employment status (ES); ^ii^= controlling for parent’s marital status (MS), CT and ES; ^iii^= controlling for MS, CT, EDU and ES; ^iv^= controlling for CT, EDU, and ES

## Table S2. Logistic regression analysis of probable PTSD predicting adolescent alcohol use among trauma–exposed Taiwanese adolescents

|  | **Han Chinese (n=263)** | | **Amis (n=228)** | | **Atayal (n=260)** | |
| --- | --- | --- | --- | --- | --- | --- |
| **Predictor** | **OR*(95% CI)** | ***P*** | **OR (95% CI)** | ***P*** | **OR (95% CI)** | ***P*** |
| **Probable PTSD**  **(reference group: no PTSD)** | | | | |  |  |
|  | 1.35 **^i^** (0.37–4.82) | 0.643 | 0.58 **^ii^** (0.18–1.86) | 0.360 | 1.74 **^i^** (0.15–19.79) | 0.654 |

*Note:* *= OR, odds ratio, CI, confidence interval; **^†^=** drinking less than twice a week in the past year**; ^§^=** drinking less than 3 times in the past year**;** ^i^=the logistic model controlling for parent’s marital status (MS), caregiver’s type, education, and employment status (ES); ^ii^= controlling for MS and ES
